# Protein Kinase C and Toll-Like Receptor Signaling

**DOI:** 10.4061/2011/537821

**Published:** 2011-08-23

**Authors:** Daniel J. Loegering, Michelle R. Lennartz

**Affiliations:** ^1^Center for Cardiovascular Sciences, Albany Medical College, 47 New Scotland Avenue, Albany, NY 12208, USA; ^2^Center for Cell Biology and Cancer Research, Albany Medical College, 47 New Scotland Avenue, Albany, NY 12208, USA

## Abstract

Protein kinase C (PKC) is a family of kinases that are implicated in a plethora of diseases, including cancer and cardiovascular disease. PKC isoforms can have different, and sometimes opposing, effects in these disease states. Toll-like receptors (TLRs) are a family of pattern recognition receptors that bind pathogens and stimulate the secretion of cytokines. It has long been known that PKC inhibitors reduce LPS-stimulated cytokine secretion by macrophages, linking PKC activation to TLR signaling. Recent studies have shown that PKC-**α**, -*δ*, -**ε**, and -**ζ** are directly involved in multiple steps in TLR pathways. They associate with the TLR or proximal components of the receptor complex. These isoforms are also involved in the downstream activation of MAPK, RhoA, TAK1, and NF-*κ*B. Thus, PKC activation is intimately involved in TLR signaling and the innate immune response.

## 1. Introduction 

Protein kinase C (PKC) is a family of protein serine/threonine kinases centrally involved in intracellular signal transduction. The PKC isoforms are divided into 3 subfamilies based on their activation requirements: the conventional isoforms, PKC-*α*, -*β*I, -*β*II, and -*γ*, require calcium, diacylglycerol, and phosphatidylserine; the novel isoforms, PKC-*δ*, -*ε*, -*η*, and –*θ*, require diacylglycerol and phosphatidylserine but are calcium independent; the atypical isoforms, PKC-*ζ* and *λ*/*ι*, require only phosphatidylserine [1]. Different isoforms of PKC are involved in such pivotal functions as cell growth, differentiation, apoptosis, motility, and secretion. Accordingly, these enzymes have been implicated in many disease states including cancer and cardiovascular disease [2–6]. The role of PKC in cancer is complicated by the tissue-specific, and often opposing, effects of the different isoforms on cell cycle and apoptosis. Similarly, the role of PKC in heart disease is complex because components of the disease (myocyte hypertrophy, cardiac function, fibrosis, and inflammation) are influenced in different ways by the different isoforms. 

Toll-like receptors (TLRs) are a family of pattern recognition receptors that are critical for the effective innate immune response to infection [7–9]. Signaling from different TLRs varies but is initiated by the recruitment of TIR-containing adaptor proteins (e.g., TIRAP recruits MyD88, TRAM recruits TRIF). MyD88 recruits IRAK4, IRAK1, IRAK2, and TRAF6. Phosphorylation and degradation of IRAK1 releases this complex into the cytoplasm where it binds and activates TAK1 downstream. Through an as yet unknown, and possibly indirect, mechanism, TAK1 activates the IKK*β* → IkB-*α* → NF-*κ*B pathway for induction of proinflammatory genes. TAK1 also activates the MAPK cascades that influence gene expression. TLR binding of TRIF recruits TRAF6, *β*RIP1, and TAK1 for activation of MAPK, IRF3, NF-*κ*B, and transcription of interferon-*β*. The TRIF pathway also stimulates the secretion of proinflammatory cytokines although to a lesser degree than the MyD88 pathway.

Initial evidence for the involvement of PKC in TLR signaling came from observations that altering PKC activity in cells of the innate immune system affected cytokine secretion. Subsequently, LPS and other TLR ligands were shown to activate most of the PKC isoforms expressed in monocytes, macrophages, dendritic cells, and neutrophils [10–14]. A large number of studies have shown that pharmacological inhibition of PKC, or its depletion by long-term treatment with phorbol esters, decreases LPS-stimulated cytokine secretion [10, 15–17]. Accordingly, acute activation of PKC with phorbol esters increases cytokine secretion [11, 15, 17, 18].

**Figure 1:**
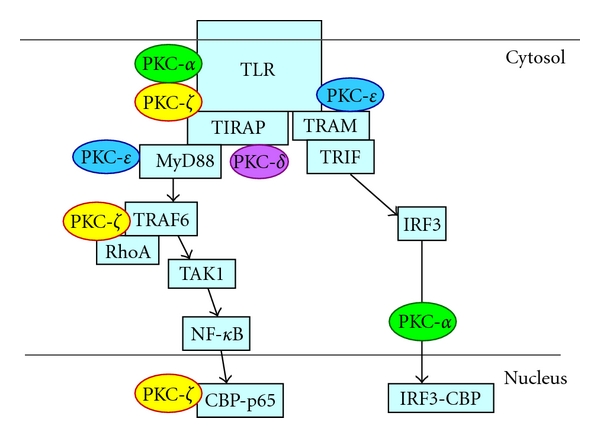
PKC isoforms act at many levels in TLR signaling. PKC-*α* associates with the TLR2 signaling complex in a MyD88-dependent manner. PKC-*α* is also required for TLR3-mediated IRF-3 binding to CBP and IFN-*β* gene induction. Interaction of PKC-*δ* with TIRAP is required for the downstream activation of NF-*κ*B. PKC-*ε* associates with complexes of MyD88 and TLR2 or TLR4 and is necessary for downstream signaling. PKC-*ε* is also required for the phosphorylation of TRAM. PKC-*ζ* is activated upon ligation of TLR2 or TLR4. During signaling by these receptors, active PKC-*ζ* binds to TLR2, associates with TRAF6 and RhoA, and is required for full transcriptional activation of p65.

Results from studies performed over the last decade have identified four PKC isoforms that impact different steps in TLR signaling. This paper will summarize recent advances detailing the role of PKC-*α*, -*δ*, -*ε*, and -*ζ* in activation of the initial TLR signaling complex, activation of RhoA, and transcription factors (Figure 1). 

## 2. PKC Isoforms Involved in TLR Signaling and Host Defense

### 2.1. PKC-*α*

Conventional PKC isoforms have been implicated in cytokine secretion by several studies showing that Gö6976, which inhibits PKC-*α* and *β*, blocks TLR-stimulated cytokine secretion by macrophages [14, 19–22]. A series of studies found that 264.7 RAW macrophages expressing a dominant negative (DN) PKC-*α* have reduced LPS-stimulated TNF-*α*, IL-1*β*, iNOS, and NF-IL6 (CAAT/enhancer-binding protein *β*) induction [23, 24]. These cells also had defects in phagocytosis, phagosome maturation, and killing of intracellular pathogens, suggesting that PKC-*α* is also involved in other, non-TLR-mediated aspects of innate immunity [25–28]. A further link between PKC-*α* and TLR-mediated responses is the finding that PPAR*γ* modulates phorbol ester-induced NF-*κ*B activation and TNF-*α* secretion by preventing the activation of PKC-*α* [18].

Findings by Langlet et al. [29] revealed that, in human DC, PKC-*α* inhibition blocked IL-12p40 secretion induced by TLR2/6, TLR 2/1, TLR5, and IL-1R, but not TLR3. The role of PKC-*α* was dissected using Gö6976, expression of DN PKC-*α*, and DC from PKC-*α* ^−/−^ mice. Using these approaches, it was determined that activation of PKC-*α* is required for TLR2/1-mediated activation of MAPK, NF-*κ*B, and AP-1 as well as secretion of TNF-*α*, IL-6, and IL-10 by DC. That immunoprecipitation of PKC-*α* from TLR2/1-activated DC captured TLR2 provides additional evidence that PKC-*α* is linked to TLR2 signaling. As TLR2 was not found in PKC-*α* immunoprecipitates of cells from MyD88^−/−^ mice suggests that MyD88 links TLR2 with PKC-*α*.

Johnson et al. [30] found that poly (I : C), a TLR3 ligand, activated PKC-*α*. Downregulation of PKC-*α* with siRNA, or expression of DN PKC-*α*, blocked TLR3-stimulated IFN-*β* production in dendritic cells (DCs). Interfering with PKC-*α* activity did not change the activation of IRF3 in terms of phosphorylation, dimerization, nuclear translocation, or DNA binding but did inhibit IRF-3 transcriptional activity induced by TRIF and TBK1 overexpression. This latter effect is due to decreased IRF-3 binding to the coactivator, CREB binding protein (CBP) which requires PKC-*α* activation. 

### 2.2. PKC-*δ*

Several studies have implicated PKC-*δ* in TLR-mediated cytokine secretion [12, 31–34]. Inhibition of PKC-*δ* with Rottlerin, or its downregulation, consistently decreases activation of NF-*κ*B, secretion of inflammatory cytokines, and production of nitric oxide by cells of the innate immune system. Results from Kubo-Murai et al. [35] suggest that PKC-*δ* is involved in TLR signaling through its interaction with TIRAP. Specifically, PKC-*δ* in macrophage lysates bound to immobilized TIRAP via the TIR domain of TIRAP. Additionally, PKC-*δ* depletion results in the loss of kinase activity in immobilized TIRAP implicating PKC-*δ* as the relevant kinase for propagating signaling from the TIRAP complex. That downregulation of PKC-*δ* severely depresses the activation of p38 MAPK and NF-*κ*B by ligands for TLR4, and TLR2 underscores its importance in signaling via these receptors. To date, this is the only report demonstrating a direct role for PKC-*δ* in TLR signaling. 

Cecal ligation and puncture (CLP) is an established animal model for sepsis and has been used to study TLR signaling *in vivo*. Recently, it was shown that, when administered intratracheally, a PKC-*δ*-inhibiting peptide reduced the lung injury associated with CLP-induced sepsis in rats [36]. This inhibitor blocked sepsis-induced phosphorylation of PKC-*δ*. Animals given the inhibitor prior to CLP had reduced levels of the chemokines CINC-1 and MIP-2 in lung lavage and blood samples. At the same time, there was reduced infiltration of inflammatory cells into the lungs and less pulmonary edema. The authors linked the protective effects of the inhibitor to reduced NF-*κ*B activation and chemokine production by macrophages, endothelial cells, and epithelial cells [37–39]. This is consistent with a previous study showing that PKC-*δ* ^−/−^ mice had reduced cytokine production, neutrophil infiltration, and lung injury due to asbestos [40].

Sphingosine kinase 1 (SphK1) has been implicated in inflammation through the formation of sphingosine 1 phosphate [41–44]. SphK1 is induced and activated by several immune stimuli including LPS. In LPS-treated macrophages, PKC-*δ* lies downstream of SphK1 activation and is required for the activation of NF-*κ*B [37]. Furthermore, the inhibition of SphK1 *in vivo* reduced LPS- and sepsis-stimulated cytokine secretion and mortality. These studies support a TLR4→SphK1→PKC-*δ*→NF-*κ*B→cytokine pathway. It remains to be determined if the same pathway links SphK1 to TNFR1 and IL-1R [43, 44]. 

Beyond the scope of this paper are the effects of this PKC isoform in several TLR-independent aspects of inflammation including signaling via TNFR1, neutrophil activation, and endothelial cell function [43–48].

### 2.3. PKC-*ε*

A role for PKC-*ε* in host defense was apparent when Castrillo et al. [49] observed that PKC-*ε* ^−/−^ mice were difficult to breed due to infections of the uterus. Additionally, these animals had a 2- to 3-fold reduction in survival time after infection with Gram-negative or Gram-positive bacteria. Several aspects of the immune system in these animals were normal although macrophages were defective in the production of LPS-stimulated TNF-*α*, IL-1*β*, PGE_2_, and nitric oxide. There were also deficits in LPS-stimulated MAPK and NF-*κ*B activation. Other studies, using PKC-*ε*-specific inhibitors, depletion with antisense oligonucleotides, and macrophages from PKC-*ε* ^−/−^ mice, verified that PKC-*ε* is critical for LPS-stimulated TNF-*α* and IL-12 secretion by DC and macrophages [10, 50, 51]. *In vivo* administration of a PKC-*ε* inhibitor reduced the inflammation associated with a murine model of cardiac transplantation [52]. Thus, PKC-*ε* has a likely role in inflammation and host defense [53, 54]. 

More recent studies have sought to clarify the role of PKC-*ε* in TLR signaling. PKC-*ε* is phosphorylated by all TLRs that signal through MyD88, that is, TLR1 through 9 except TLR3 in macrophages [55]. Upon TLR4 ligation with LPS, PKC-*ε* is phosphorylated on Ser-346 and 368 and binds to 14-3-3*β*. Association with 14-3-3*β* requires the presence of MyD88. Phosphorylation of these serines is critical for PKC-*ε* signaling as cells expressing PKC-*ε* S346A/S368A fail to activate an NK-*κ*B reporter in response to ligands for TLR4 and TLR2. These findings suggest that the complex of TLR, MyD88, 14-3-3*β*, and PKC-*ε* is required for gene induction. Of note, since PKC-*ε* can be phosphorylated by PKC-*α*, it is possible that the effects of PKC-*α* on cytokine secretion are shared by PKC-*ε* [56, 57]. 

In addition to its role in MyD88-dependent signaling, PKC-*ε* is also involved in TLR4 activation via the TRAM pathway. The TRAM pathway primarily stimulates the production of IFN-*β* and RANTES. Phosphorylation of TRAM in LPS-stimulated macrophages allows it to dissociate from the membrane and bridge TLR4 with TRIF. McGettrick et al. [11] found that recombinant PKC-*ε*, but not PKC-*ζ*, phosphorylates TRAM on Ser-16, and TRAM^−/−^ macrophages reconstituted with TRAM S16A do not signal. That this phosphorylation of TRAM did not occur in PKC-*ε* ^−/−^ cells and that macrophages from PKC-*ε* ^−/−^ mice have reduced production of IFN-*β* places PKC-*ε* in the TLR4→ PKC-*ε* → Ser-16 TRAM→ IFN-*β* pathway. 

### 2.4. PKC-*ζ*

This atypical PKC isoform is a component of the signaling pathways for IL-1R and TNFR [58–60]. More recently, PKC-*ζ* has been shown to be involved in the activation of TLR, IRAK, RhoA, and NF-*κ*B [61–64]. 

PKC-*ζ* is downstream of TLR2 in human macrophages stimulated with* Mycobacterium tuberculosis* (MTB) [64]. Specific inhibitors of PKC-*ζ* or its downregulation blocked ERK activation and TNF-*α* secretion stimulated by MTB. PKC-*ζ* was also found to associate with immunoprecipitated TLR2 but not TLR4 after stimulation with MTB or peptidoglycan. Finally, PKC-*ζ* did not associate with TLR2 in THP-1 cells expressing DN PKC-*ζ*. These results suggest a TLR2 → PKC-*ζ*→ ERK pathway for production of TNF-*α* in response to MTB. 

IRAK phosphorylation is an early event in TLR signaling. Phosphorylated IRAK is subsequently degraded which acts as a negative feedback control on the signaling pathway. Using a panel of protein kinase inhibitors, Hu et al. [61] demonstrated that the phosphorylation of IRAK by TLR4 is mediated by PKC in THP-1 cells. PKC-*ζ* was found in IRAK immunoprecipitates from LPS-stimulated cells. Furthermore, macrophages from wild-type mice responded to LPS with activation of PKC-*ζ* and phosphorylation of IRAK, but macrophages from C3H/HeJ mice that have a nonfunctional TLR4 did not. Although not yet directly tested, these results suggest that IRAK is a PKC-*ζ* substrate. 

Several studies have highlighted a role for RhoA in TLR signaling [65–69]. Two papers have directly implicated PKC-*ζ* in TLR2 and TLR4 signaling through the activation of RhoA and subsequent activation of NF-*κ*B [62, 63]. Teusch et al. [63] found that full transcriptional activation of p65 by TLR2 ligands in monocytic cells requires the activity of both RhoA and PKC-*ζ*. Inhibition of PKC-*ζ*, or expression of its dominant negative mutant, reduces the phosphorylation of p65 on serine 311 and its transcriptional activity. PKC-*ζ* transiently associates with RhoA with a time course similar to that of RhoA activation. The authors concluded that PKC-*ζ* mediates at least part of the effects of RhoA on gene transcription. 

Further refinement of the position of PKC-*ζ* in the TLR→ RhoA → NF-*κ*B pathway has been elucidated by the studies of Huang et al. [62]. They reported that treatment of macrophages with LPS activates PKC-*ζ* and that interfering with PKC-*ζ* activation blocks LPS-stimulated activation of NF-*κ*B and cytokine production. As in the TLR2 studies above, PKC-*ζ* was present in anti-RhoA or anti-TRAF6 immunoprecipitates of cell lysates from LPS-stimulated macrophages. Inhibition of RhoA or TRAF6 blocked PKC-*ζ* activation, placing PKC-*ζ* downstream of RhoA and TRAF6. Similar experiments place TAK1 between PKC-*ζ* and NF-*κ*B. That is, inhibition of PKC-*ζ* blocked TAK1 phosphorylation, and constitutively active PKC-*ζ* fails to activate NF-*κ*B in TAK1-depleted cells. Together, these studies are consistent with the following model: TLR2/4→ RhoA/TRAF6→ PKC-*ζ*→ TAK1 → s311 p65 → cytokine induction. 

Cigarette smoke is a major cause of lung inflammation which is exacerbated by its common contamination with LPS. Yao et al. [70] recently demonstrated that PKC-*ζ* ^−/−^ mice exposed to smoke/LPS had less lung inflammation than wild-type mice. Bronchoalveolar cells from mice exposed to smoke/LPS had activated PKC-*ζ* that translocated to the nucleus in association with p65 and CBP. This was associated with the phosphorylation (Ser311) of p65 and induction of cytokines. All of these events were reduced in PKC-*ζ* ^−/−^ mice. 

Surfactant protein A (SP-A) limits several aspects of inflammation in the lung, partly due to stabilization of IkB-*α* [71]. Moulakakis et al. [72] found that PKC-*ζ* is required for this effect. SP-A activates PKC-*ζ*, limits its translocation to the nucleus, and stabilizes IkB-*α*. In alveolar macrophages from PKC-*ζ* ^−/−^ mice, these protective effects were lost. That is, in PKC-*ζ* ^−/−^ macrophages, SP-A failed to inhibit LPS-stimulated IkB-*α* activation, and NF-*κ*B DNA binding was nearly normal. It was concluded that the effect of SP-A → PKC-*ζ* axis may function as a brake on the inflammatory response of alveolar macrophages. 

## 3. Perspective

The establishment of PKC in TLR signaling as well as in other inflammatory processes may provide a means to treat inflammatory diseases. Inhibition of a kinase required for so many important biological functions may be expected to result in substantial side effects. However, several inhibitors of PKC have proven to be well tolerated in clinical trials [73–75].

The studies detailed above provide convincing evidence that PKC is directly involved in TLR signaling. In most cases, further studies are needed to determine the nature of the interaction between PKC isoforms and other proteins in the signaling cascades and then to identify the specific PKC substrates. Another important area of investigation is the integration of the TLR→PKC axis with the involvement of these same isoforms (PKC-*α*, -*δ*, and -*ζ*) and possible interaction of the isoforms in non-TLR-dependent aspects of innate immunity. PKC-*α* may contribute to phagocytosis and pathogen killing [25–28], PKC-*δ* is involved in signaling via TNFR1, neutrophil activation, and endothelial cell function [43–48], and PKC-*ζ* participates in signaling through TNFR and IL-1R [58–60]. Thus, PKC isoforms likely sit at different nodes in the signaling web. Understanding their contribution to individual pathways as well as their position in the web is necessary to avoid misinterpreting results of experiments such as one in which a PKC inhibitor appears to reduce the TNF-*α* response to a TLR ligand, but, in fact, the inhibitor blocked the secondary augmentation of TNF-*α* secretion mediated through the TNFR. Finally, understanding the role of TLR-mediated activation of specific PKC isoforms will immediately provide insights into the mechanisms of the disease.

One of the more important advances during the 20th century was the alleviation of a great deal of the suffering from infectious diseases. We may hope for a similar advance with regard to inflammatory diseases in the 21st century.
